# Impact on the Gut Microbiota of Intensive and Prolonged Antimicrobial Therapy in Patients With Bone and Joint Infection

**DOI:** 10.3389/fmed.2021.586875

**Published:** 2021-03-05

**Authors:** Benoît Levast, Nicolas Benech, Cyrielle Gasc, Cécile Batailler, Eric Senneville, Sébastien Lustig, Cécile Pouderoux, David Boutoille, Lilia Boucinha, Frederic-Antoine Dauchy, Valérie Zeller, Marianne Maynard, Charles Cazanave, Thanh-Thuy Le Thi, Jérôme Josse, Joël Doré, Frederic Laurent, Tristan Ferry

**Affiliations:** ^1^MaaT Pharma, Lyon, France; ^2^Service des Maladies Infectieuses et Tropicales, Hôpital de la Croix-Rousse, Hospices Civils de Lyon, Lyon, France; ^3^Université Claude Bernard Lyon 1, Lyon, France; ^4^Centre de Référence Pour la Prise en Charge des Infections Ostéo-Articulaires Complexes (CRIOAc Lyon), Hospices Civils de Lyon, Lyon, France; ^5^TGF-ß Immune Evasion, Tumor Escape Resistance Immunity Department, Cancer Research Center of Lyon, Inserm 1052, CNRS 5286, Lyon, France; ^6^Service de Chirurgie Orthopédique, Hôpital de la Croix-Rousse, Hospices Civils de Lyon, Lyon, France; ^7^Service de Maladies Infectieuses et du Voyageur, Centre Hospitaliser Gustave Dron, Tourcoing, France; ^8^Université de Lille, Lille, France; ^9^Centre de Référence Pour la Prise en Charge des Infections Ostéo-Articulaires Complexes (CRIOAc Lille-tourcoing), Tourcoing, France; ^10^Service de Maladies Infectieuses et Tropicales, Hôpital de l'Hôtel-Dieu, CHU de Nantes, Nantes, France; ^11^Université de Nantes, Nantes, France; ^12^Centre de Référence des Infections Ostéo-Articulaires Grand-Ouest, Nantes, France; ^13^Centre Hospitalier Universitaire de Bordeaux, Service des Maladies Infectieuses et Tropicales, Hôpital Pellegrin, CHU de Bordeaux, Centre de référence des Infections Ostéoarticulaires Complexes du Grand Sud-Ouest (CRIOAc GSO), Bordeaux, France; ^14^Service de Médecine Interne et Rhumatologie, GH Diaconesses-Croix Saint-Simon, Paris, France; ^15^Centre de Référence Infections Ostéoarticulaires Complexes de Paris (CRIOAc Paris), Paris, France; ^16^Centre de Recherche Clinique, Hôpital de la Croix-Rousse, Hospices Civils de Lyon, Lyon, France; ^17^Univ. Bordeaux, USC EA 3671, Infections Humaines à Mycoplasmes et à Chlamydiae, Bordeaux, France; ^18^Centre de Ressource Biologique, Hôpital de la Croix-Rousse, Hospices Civils de Lyon, Lyon, France; ^19^Institut des Agents Infectieux, Laboratoire de Bactériologie, Hôpital de la Croix-Rousse, Hospices Civils de Lyon, Lyon, France; ^20^CIRI – Centre International de Recherche en Infectiologie, Inserm, U1111, Université Claude Bernard Lyon 1, CNRS, UMR5308, Ecole Normale Supérieure de Lyon, Univ Lyon, Lyon, France; ^21^Université Paris-Saclay, INRAE, MetaGenoPolis, AgroParisTech, MICALIS, Jouy-en-Josas, France

**Keywords:** gut microbiota, antimicrobial therapy, antibiotics, bone and joint infection, dysbiosis

## Abstract

There is a growing interest in the potentially deleterious impact of antibiotics on gut microbiota. Patients with bone and joint infection (BJI) require prolonged treatment that may impact significantly the gut microbiota. We collected samples from patients with BJI at baseline, end of antibiotics (EOT), and 2 weeks after antibiotic withdrawal (follow-up, FU) in a multicenter prospective cohort in France. Microbiota composition was determined by shotgun metagenomic sequencing. Fecal markers of gut permeability and inflammation as well as multi-drug-resistant bacteria (MDRB) and *Clostridioides difficile* carriage were assessed at each time point. Sixty-two patients were enrolled: 27 native BJI, 14 osteosynthesis-related BJI, and 21 prosthetic joint infections (PJI). At EOT, there was a significant loss of alpha-diversity that recovered at FU in patients with native BJI and PJI, but not in patients with osteosynthesis-related BJI. At EOT, we observed an increase of Proteobacteria and Bacteroidetes that partially recovered at FU. The principal component analysis (PCoA) of the Bray–Curtis distance showed a significant change of the gut microbiota at the end of treatment compared to baseline that only partially recover at FU. Microbiota composition at FU does not differ significantly at the genus level when comparing patients treated for 6 weeks vs. those treated for 12 weeks. The use of fluoroquinolones was not associated with a lower Shannon index at the end of treatment; however, the PCoA of the Bray–Curtis distance showed a significant change at EOT, compared to baseline, that fully recovered at FU. Levels of fecal neopterin were negatively correlated with the Shannon index along with the follow-up (*r*^2^ = 0.17; *p* < 0.0001). The PCoA analysis of the Bray–Curtis distance shows that patients with an elevated plasma level of C-reactive protein (≥5 mg/L) at EOT had a distinct gut microbial composition compared to others. MDRB and *C. difficile* acquisition at EOT and FU represented 20% (7/35) and 37.1% (13/35) of all MDRB/*C. difficile*-free patients at the beginning of the study, respectively. In patients with BJI, antibiotics altered the gut microbiota diversity and composition with only partial recovery, mucosal inflammation, and permeability and acquisition of MDRB carriage. Microbiome interventions should be explored in patients with BJI to address these issues.

## Introduction

Bone and joint infections (BJI) are a public health issue in industrialized countries (1). Different kinds of BJI exist, depending on the pathophysiology and the route of bone contamination. BJI may occur spontaneously, such as hematogenous spondylodiscitis, septic arthritis, or diabetic foot infection with osteomyelitis, and these BJI can be grouped as “native BJI” (2, 3). Osteosynthesis-associated BJI frequently involves the long bones, especially the tibia. Fracture-related infections are major contributors to this group of BJI (4). Prosthesis joint infection (PJI) is the last group of BJI that mainly occurs in an elderly population in whom a hip or a knee prosthesis becomes infected after its implantation (5). Whatever the mechanism of acquisition, a BJI is considered as one of the most difficult-to-treat bacterial infections, as the eradication of the pathogen is challenging (1). The multidisciplinary management and a medico-surgical approach are crucial in all these patients. Surgery is essential in most of them, and upon diagnosis of BJI immediately after surgery, an intravenous, broad-spectrum empirical antimicrobial therapy is usually started and secondarily adapted to the microbiological culture results that reveal the pathogens involved and their susceptibilities to antibiotics. Depending on the type of BJI and the clinical presentation, oral antibiotics are frequently prolonged for a total of 6 or 12 weeks of therapy (2, 3, 5, 6). As it is one of the longest duration of antibiotics for a bacterial infection, it could be associated with significant side effects, such as a huge impact on the gut microbiota and the promotion of acquired antimicrobial resistance (7–9).

It has been demonstrated that short-term antibiotic usage strongly affects the gut microbiota. Extensive literature describing alteration of the gut microbiota by the use of antibiotics has bloomed over the recent years (10–13). The most frequent manifestation is antibiotic-associated diarrhea (14), which may be due to the direct toxin effects of antibiotics on the intestine, altered digestive function secondary to reduced concentrations of gut bacteria (“functional diarrhea”), or overgrowth of pathogenic microorganisms, such as *Clostridioides difficile* infection. The latter can account for most of the severe antibiotic-associated diarrhea observed and is characterized by a loss of the gut microbiota barrier properties, leading to frequent *C. difficile* infection recurrence with high morbidity. Of note is that one of the most effective treatments of recurrent *C. difficile* infection is fecal microbiota transplantation, which aims at restoring the fecal microbial ecosystem by transferring into the gut a preparation of feces from a healthy donor (15).

Nowadays, the gut microbiota is considered a major factor involved in the pathophysiology of many diseases. The development of novel molecular technologies coupling high-throughput metagenomic sequencing and bioinformatics/biostatistics has overcome the limitation of culture-based analysis of stool samples, opening a new way for the exploration of the gut microbiota in various diseases such as inflammatory bowel diseases (16), metabolic disorders (17), infectious diseases (18), or, more recently, central nervous system diseases (19). In all these conditions, an altered composition of the gut microbiota has been described, suggesting the deleterious effect on host physiology of modified gut microbial communities.

Infections and the use of antibiotics can cause significant and sometimes irreversible effects on the gut microbial composition throughout life (20, 21). The manner in which antibiotics affect gut microbial communities can vary according to different parameters: the route of antibiotic administration (22, 23), the duration of treatment (24), the broad or narrow spectrum of action of bacterial species-targeted antibiotics (25), the use of antibiotic combinations, or the repetition of antimicrobial treatment (26). The impact caused by antibiotics on gut microbiota results in alteration of the bacterial diversity and gut functions. These alterations could be transitory or could last over time (21). After antibiotic withdrawal, the gut microbiota has, in theory, the potential ability to return to its base state by a mechanism called resilience (26). However, resilience after prolonged antibiotic exposure has been poorly evaluated.

Antibiotics and gut microbiota modifications have also been associated with local alterations in gut physiology with inflammation and increased permeability (27). To explore these aspects within the clinical model of BJI treatment, we assessed the dynamics of various markers of intestinal inflammation such as fecal neopterin and calprotectin, which are increased in an inflammatory context (28, 29) and zonulin, the only known protein that regulates intercellular tight junctions (30).

The main concern with prolonged antibiotic treatment is the emergence of antimicrobial resistance and its spread into a patient's environment (31). Extensive use of antibiotics in the last 40 years has systematically led to the emergence of bacterial resistance and development of nosocomial infections, mainly due to methicillin-resistant *Staphylococcus aureus* (MRSA) in the 1990s and now particularly due to commensals of the gut microbiota such as multidrug-resistant (MDR) Enterobacteriaceae. These hospital-acquired infections are causes of considerable morbidity and mortality in many industrialized countries and are one of the major concerns of public health issues and threats. In the last 10 years, MDR Enterobacteriaceae, particularly MDR *Escherichia coli* and *Klebsiella pneumoniae*, also emerged as community-acquired infections (i.e., infections contracted outside of a healthcare setting) (32, 33). Antibiotic treatments may facilitate the acquisition of MDR bacteria (MDRB) in the gut that can disseminate in the patient's environment (31).

In this study, we aimed to investigate for the first time the microbiological, clinical, and biological consequences on the gut microbiota and its host of prolonged antibiotic treatment in patients with different types of BJI. Antibiotic-related gut microbiota modifications and its resilience were assessed using shot-gun metagenomic sequencing after different treatment durations (6 weeks compared to 12 weeks) and different types of antibiotics, while potential association of the gut microbiota composition was investigated with (i) gut markers of inflammation and permeability, (ii) C-reactive protein (CRP), a systemic marker of inflammation that usually followed during BJI, and (iii) the acquisition of MDRB or *C. difficile* fecal carriage.

## Materials and Methods

### Study Description

We performed a multicentric prospective interventional study in France called Interventi*O*nal *S*tudy of Bone and Joint *I*nfections *R*elated Gut Dysbios*IS* (OSIRIS) (NCT03011502; EudraCT 2016-003247-10) from January 2017 to September 2017. Five recruiting centers belonging to the CRIOAc network, a nationwide network of clinical centers dedicated to the management of complex BJI, were selected: Lyon (Hospices Civils de Lyon, CRIOAc Lyon), Bordeaux (CRIOAc GSO), Nantes (CRIOGO), Paris (CRIOAc Paris), and Lille-Tourcoing (CRIOAc G4).

### Ethics

Patients highly suspected of BJI were informed and enrolled after their signature of written consent in the OSIRIS protocol. Patient follow-up was designed as part of standard care, with some interventions dedicated to the specific needs of the protocol. This study was reviewed and approved by a regional ethics committee (Comité de Protection des Personnes SUD-EST II; 69HCL16_0623). The study was also approved by the French Health authority (Agence Nationale de Sécurité du Médicament et des produits de Santé, ANSM).

### Data Collection

Clinical data and stool collection were performed at baseline visit (B) within 24 h before starting the antibiotics, at the end of the treatment (EOT), and at 2 weeks after antibiotic withdrawal during a follow-up visit (FU). An electronic case report form (e-CRF) was created, and clinical data and the results of the serum CRP measurements were prospectively collected during follow-up using the ClinSight™ software.

Data that support the findings of the study are available from the corresponding author upon reasonable request.

### Stool Collection and Fecal Microbiome Analysis

The patients collected their stools using a dedicated clean container system, ensuring stool conservation (Fecotainer®) within 48 h prior to freezing. The samples were then snap-frozen in triplicates of 1 g and stored at−80°C. At the end of the follow-up of the last patient, the samples were sent to Eurofins Inc., for DNA extraction and shotgun analysis. Genomic DNA was extracted from the fecal samples using the Qiagen QIAamp Fast DNA stool mini-kit after bead beating. Positive (*Escherichia coli*) and negative (no template, i.e., water) controls have been added throughout the process, from DNA extraction to sequencing, to validate the successful completion of each step. Sequencing library was constructed for each DNA sample using the TruSeq Nano DNA Library Prep kit (Illumina) according to the manufacturer's instructions. Libraries were then sequenced in paired-end (2 × 125 bp) HiSeq2500 v4 (Illumina) runs.

Bioinformatics analyses were performed on the Gut Print® platform with the in-house MgRunner v1.1.2 pipeline. In brief, after quality filtering using Trimmomatic (34), host sequence removal was performed using Bowtie2 (35). To ensure comparability, all samples were rarefied to the same sequencing depth, i.e., 5,000,000 paired-end sequences per sample. Taxonomic profiling was then performed with Kraken v.0.10.5-beta (36) and the RefSeq genomic database (2015 release, http://www.ncbi.nlm.nih.gov/refseq/). The measurement of α- and β-diversity indexes was performed in R Statistical Software ((37), version 3.4.4, http://www.R-project.org) using vegan and phyloseq packages. Identification of marker taxa for the different groups was achieved through differential abundance analysis using linear discriminant analysis effect size (38).

#### Neopterin, Calprotectin, Zonulin, and IgA Quantification in Stools

Biological markers of gut permeability and inflammation were monitored at each time point. At the end of the follow-up of the last patient, the samples were sent to the biochemistry laboratory of the HCL Centre Hospitalier Lyon Sud for ELISA-related techniques of analysis to be performed. Supernatants were obtained in the laboratory and then run to quantify sIgA (ImmunoChrom kit RIC6100—BioVendor), neopterin (kit neopterin ELISA Ref59321—IBL International), calprotectin (fCAL RefEKCAL2—Bülhmann), and zonulin (RefK5600—ImmunDiagnostik), according to the manufacturer's instructions.

### Fecal MDRB Cultures and Antibiogram Analysis

Fecal swabs were sampled at each specified patient visit and then sent prospectively to the IAI for fresh cultures on specific gelose media to evaluate the fecal portage of MDRB such as extended-spectrum beta-lactamases-producing Enterobacteriaceae (ESBL), carbapenemase-producing Enterobacteriaceae, MRSA, and *C. difficile*. When positive (i.e., detection of a growth) in selective chromogenic media (ChromID?: MRSA, ESBL, OXA48 and CARBA SMART, Biomérieux, Marcy-l'étoile, France), antibiograms and mass spectrometry were done on isolates to specify the minimum inhibitory concentrations of specific antibiotics and specify the isolates' identification (genus/species). When positive for ESBL, patient samples were thawed to quantify ESBL and total Gram-negative bacteria.

### Statistical Analysis

Linear regression, Wilcoxon, and Mann–Whitney test were performed with Prism (GraphPad®, version 8.4.3, GraphPad Software, La Jolla California, USA; www.graphpad.com). *P* values are indicated in the graphs unless specified in the legend. Permutational multivariate analysis of variance (PERMANOVA) was performed with the R Statistical Software using the Adonis package. Analyses were performed both at the genus and OTU levels with no qualitative differences between the two taxonomic levels.

## Results

### BJI Population

**Figure 1:**
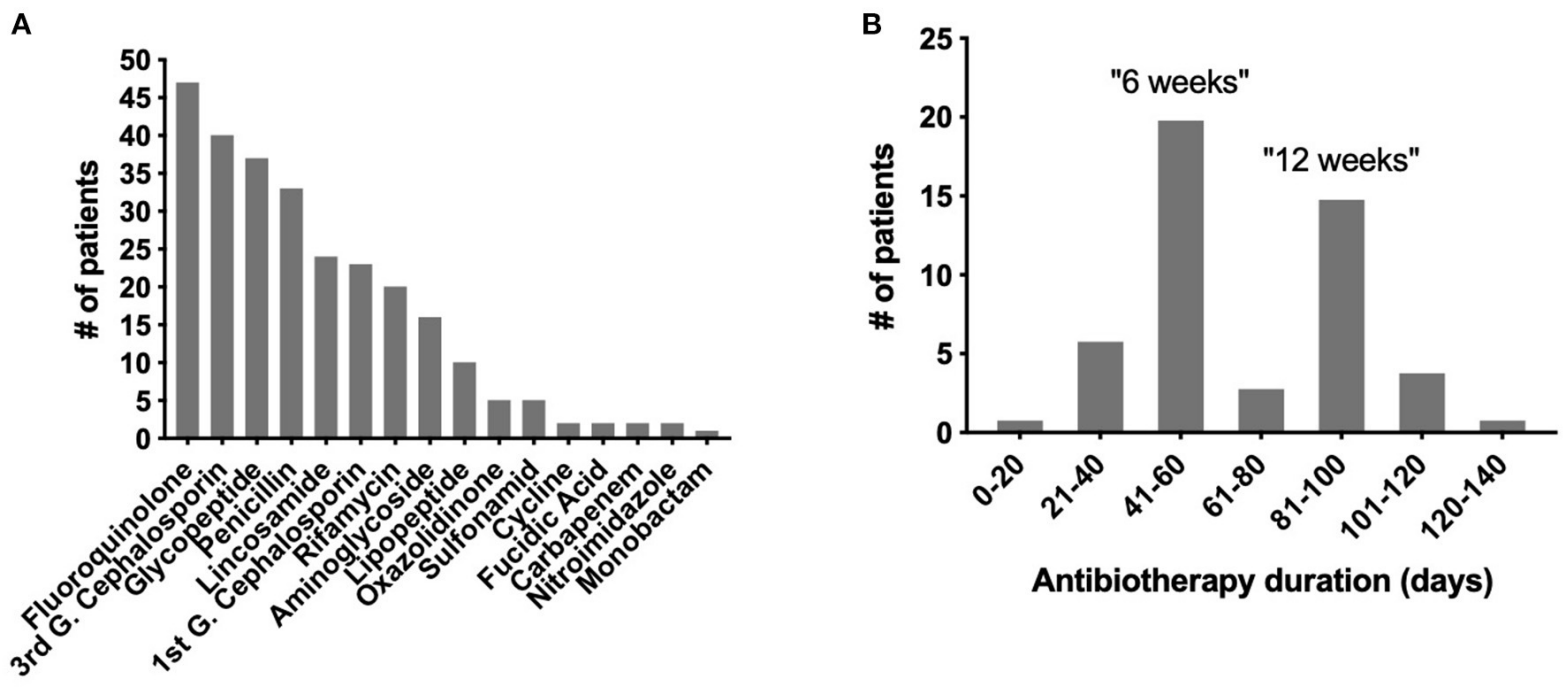
Types of antibiotics and treatment duration in the OSIRIS cohort. **(A)** Distribution of the different types of antibiotics used. **(B)** Distribution of the antibiotic durations used.

At the end of the inclusion period, 62 patients were enrolled, including 27 with a native BJI, 14 with an osteosynthesis-related BJI, and 21 with a PJI. The patients' characteristics are detailed in Table 1, and the patients' characteristics during the study are presented in Table 2. Each patient received personalized antimicrobial therapy, from empirical to targeted treatment, depending on the microbiological culture results and local and recommended treatment strategy. A total of 16 different types of antibiotic were used. The most frequent one was fluoroquinolone (FQ) (*n* = 47), a category that includes ofloxacin, levofloxacin, and ciprofloxacin (Figure 1A). After excluding extreme values, the duration of antimicrobial treatment was divided into two different groups corresponding to the two mainly recommended antibiotic duration in BJI: a “6 weeks” group (*n* = 20; between 41 to 60 days) and a “12 weeks” group (*n* = 15; between 81 and 100 days; Figure 1B).

**Table 1:** Patients' characteristics.

**BJI** **population**	**Native BJI** **(*n* = 27)**	**Osteosynthesis-related BJI** **(*n* = 14)**	**PJI** **(*n* = 21)**	**Total** **(*n* = 62)**
Male (*n*, %)	17 (63)	10 (71.5)	13 (62)	40 (64.5)
Age (years)^a^	56.1 (13.2)	51.8 (17.6)	65.3 (9.1)	58.6 (14.1)
Antibiotic duration (days)^a^	58.8 (26.7)	69.8 (28.4)	68.3 (29.3)	64.5 (27.8)
BMI (mean)^a^	25.6 (6.5)	28.1 (5.8)	29.5 (7.0)	27.5 (6.6)
MDRB carriage at baseline (*n*, %)				
- MRSA	3 (11.1)	1 (7.1)	5 (23.8)	9 (14.5)
- ESBL-producing	0	0	0	0
- Enterobacteriaceae	3 (11.1)	1 (7.1)	5 (23.8)	9 (14.5)
- HREB	0	0	0	0
*Clostridioides difficile* carriage at baseline (*n*, %)	1 (3.7)	0	0	1 (1.6)

a *Data are expressed as mean (standard deviation)*.

*BJI, bone joint infection; PJI, prosthesis joint infection; BMI, body mass index; MDRB, multidrug-resistant bacteria; MRSA, methicillin-resistant Staphylococcus aureus; ESBL, extended-spectrum beta-lactamases; HREB, highly resistant emergent bacteria*.

**Table 2:** Patients' characteristics along the study.

**BJI population**	**Baseline**	**End of treatment**	**Follow-up**	**Normal values**
Feces weight (g)	60.5 (*n* = 54)	61.5 (*n* = 46)	72 (*n* = 46)	100–200
MDRB fecal carriage (%)	17.54	25.00	39.13	NA
DNA extraction	1.04 ± 0.14	0.69 ± 0.12	0.84 ± 0.14	NA
Neopterin^a^ (pmol/g)	97.7	494.7^b^	285.4^b^	55
Calprotectin^a^ (μg/g)	128.6	49.4^b^	53.5^b^	<50
Zonulin^a^ (ng/mL)	85.2	128.1^b^	131.0^b^	61 ± 46
IgA^a^ (μg/g)	2,187.6	2,235.5	2,204.7	2,000
CRP^c^ (mg/L)	50.3	6	6.6	<5

a *Quantification in fecal supernatants*.

b *Significant p value <0.05, Wilcoxon test*.

c *Blood quantification*.

*BJI, bone and joint infection; MDRB, multidrug-resistant bacteria; IgA, immunoglobulin A; CRP, C-reactive protein; NA, not applicable*.

### Antibiotics Alter Gut Microbiota Diversity and Composition With Partial Recovery 2 Weeks After Antibiotic Withdrawal

**Figure 2:**
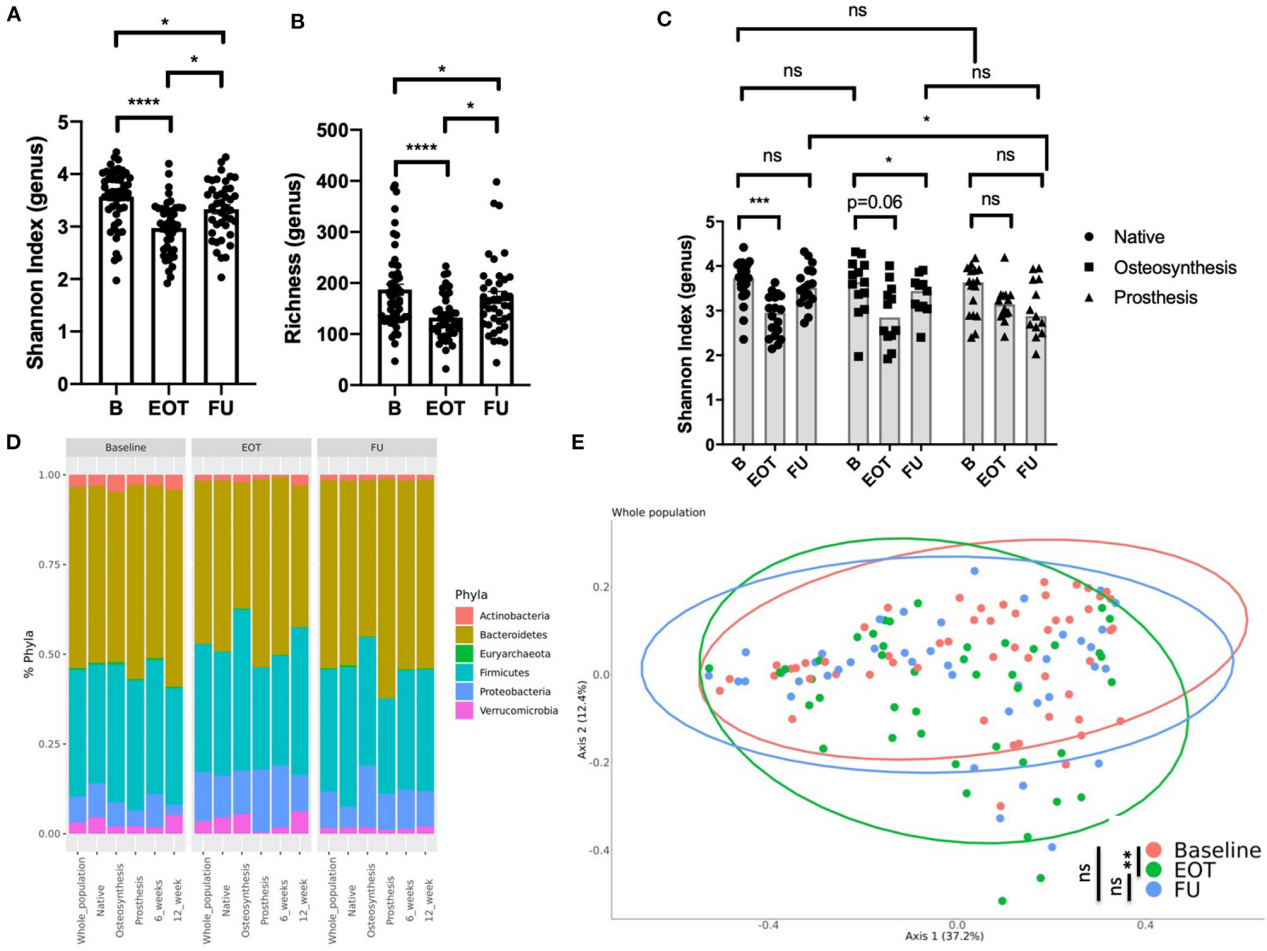
Effect of antibiotics on the gut microbiota in bone joint infection (BJI). **(A)** Shannon index (genus level) at three times of sampling; Wilcoxon test. **(B)** Richness (genus level) at three times of sampling; Wilcoxon test. **(C)** Shannon index at three times of sampling according to the type of BJI; Wilcoxon test for paired comparison, Mann–Whitney analysis for inter-group comparisons. **(D)** Global composition of bacterial microbiota at the phylum level. **(E)** Beta diversity. Principal coordinate analysis of Bray–Curtis distance at three different times of sampling; permutational multivariate analysis of variance. B, baseline; EOT, end of treatment; FU, follow-up. *p < 0.05; **p < 0.01; ***p < 0.001; ****p < 0.0001.

BJI antimicrobial therapies alter the gut microbiota diversity as demonstrated by a decrease of the Shannon index and richness between B and EOT (Figures 2A,B). However, after 2 weeks of antibiotic withdrawal, the microbiota diversity increased but remained at lower values than those before treatment (Figures 2A,B). Interestingly, resilience differed between the three different subpopulations of BJI (Figure 2C and Supplementary Figure 1). Indeed native BJI showed no significant difference between B and FU for the Shannon Index and richness, whereas osteosynthesis-related BJI presented only a partial recovery at FU for both parameters. Patients with PJI presented a lower Shannon index at FU (mean = 3.0 ± 0.6) compared to EOT (mean = 3.2 ± 0.4) and B (mean = 3.6 ± 0.6), even if it did not reach statistical significance probably because of the small sample size (Figure 2C). Interestingly, when considering the gut microbiota composition using taxonomic analysis, we observed an increase of Proteobacteria that partially recovered at FU (relative abundance at B: 7.2%, EOT: 13.5%, and FU: 10.2%).

In PJI, there was also a decrease of Firmicutes relative abundance compared to baseline (relative abundance at B: 36.1%, EOT: 28.4%, and FU: 26.5%; Figure 2D). Considering the whole population of BJI, the principal coordinate analysis (PCoA) of the Bray–Curtis distance showed significant changes of the gut microbiota at the end of treatment that only partially recovered at FU (Figure 2E). The compilation of the 20 bacteria that varied the most in relative abundance between B and EOT and B and FU for each group of patients is presented in Supplementary Figures 2A,B, respectively. A linear discriminant analysis effect size showing species that supported the differences between baseline and EOT is also shown Supplementary Figure 3. As PJI usually concern older patients for whom the microbiota may show a lower alpha-diversity, we evaluated if the age at baseline could be correlated with the Shannon index at different times of treatment. No correlation was found between age and Shannon index at B, EOT, and FU nor between body mass index and Shannon index (Supplementary Figure 4).

### No Difference of Gut Microbiota Diversity and Recovery After Antibiotic Withdrawal Between 6 and 12 Weeks of Treatment

**Figure 3:**
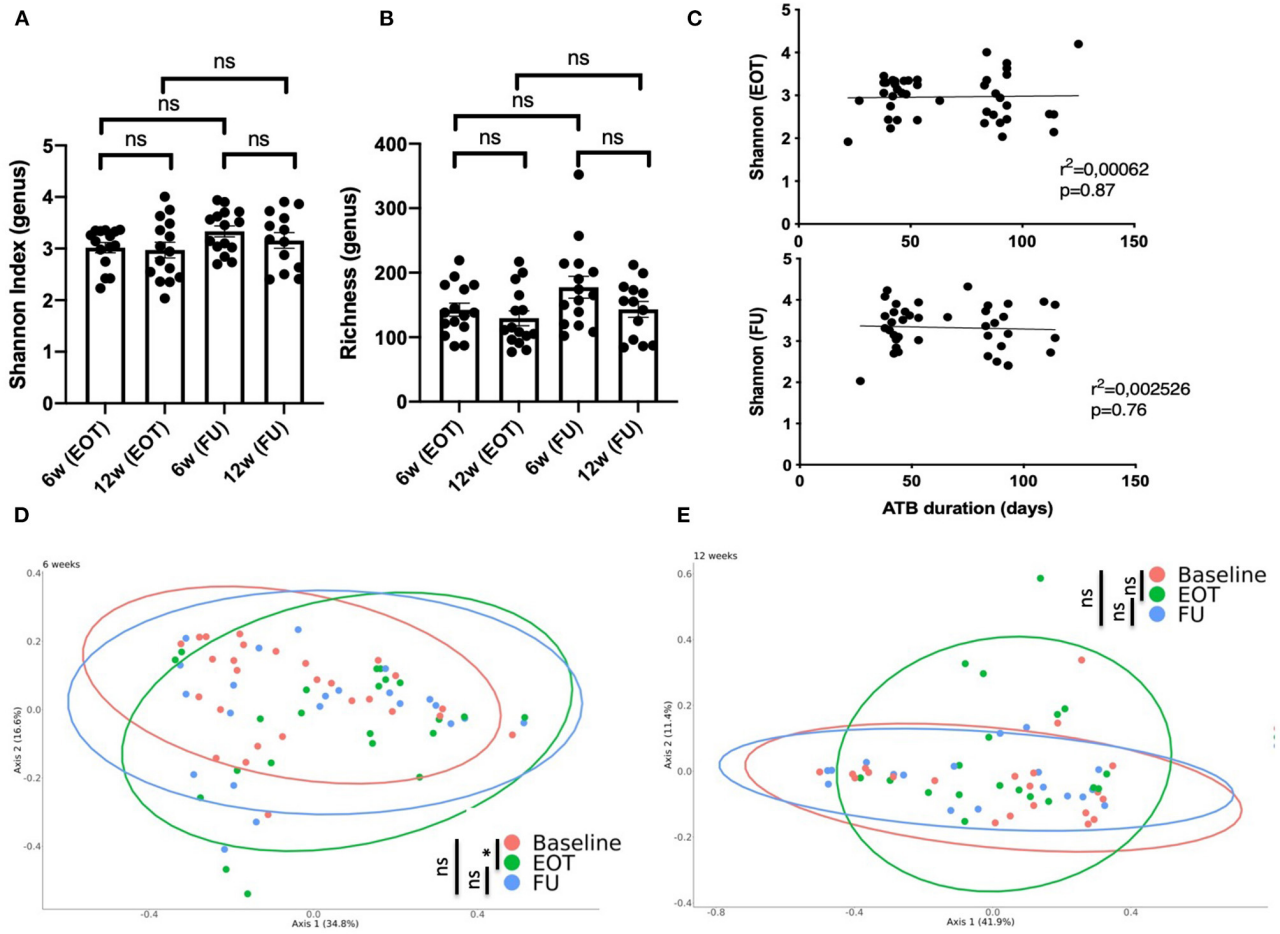
Impact of antibiotic duration on the gut microbiota composition. **(A)** Shannon index distribution (genus level) at the end of treatment and at follow-up (15 days after antibiotic withdrawal) according to antibiotic duration; Wilcoxon test for paired comparison and, Mann-Whitney test for inter-group comparisons. **(B)** Richness distribution (genus level) at the end of treatment and at follow-up (15 days after antibiotic withdrawal) according to antibiotic duration; Wilcoxon test for paired comparison and, Mann-Whitney test for inter-group comparisons. **(C)** Correlation between the Shannon index and antibiotic duration at EOT and FU; simple linear regression. Principal coordinate analysis of Bray–Curtis distance at three different times of sampling for **(D)** patients treated for 6 weeks with antibiotics (41 to 60 days) or **(E)** 12 weeks with antibiotics (81 to 100 days); permutational multivariate analysis of variance. B, baseline; EOT, end of treatment; FU, follow-up. *p < 0.05; **p < 0.01; ***p < 0.001.

Taxonomic analysis showed that the microbiota composition at FU did not differ significantly at the phylum level when comparing patients treated for 6 and 12 weeks with antibiotics (Figure 2D). Considering alpha-diversity, there was no significant difference between 6 and 12 weeks at EOT and FU (Figures 3A,B). Moreover, the Shannon index at EOT and FU did not correlate with antibiotic duration (Figure 3C). Accordingly, the PCoA of the Bray–Curtis distance showed that there was no difference in terms of recovery when comparing FU to baseline, whether antibiotic withdrawal occurred after 6 or 12 weeks of treatment (Figures 3D,E). However, analysis at the species level showed actual differences underlying potentially distinct pathophysiological functions (Supplementary Figure 2). Of note is the fact that no difference was found when comparing the composition of the gut microbiota for 6 and 12 weeks of treatment at any timepoint (data not shown).

### Treatment by Fluoroquinolone Was Associated With Significant Changes of Gut Microbiota Composition That Rapidly Recover After Antibiotic Withdrawal

**Figure 4:**
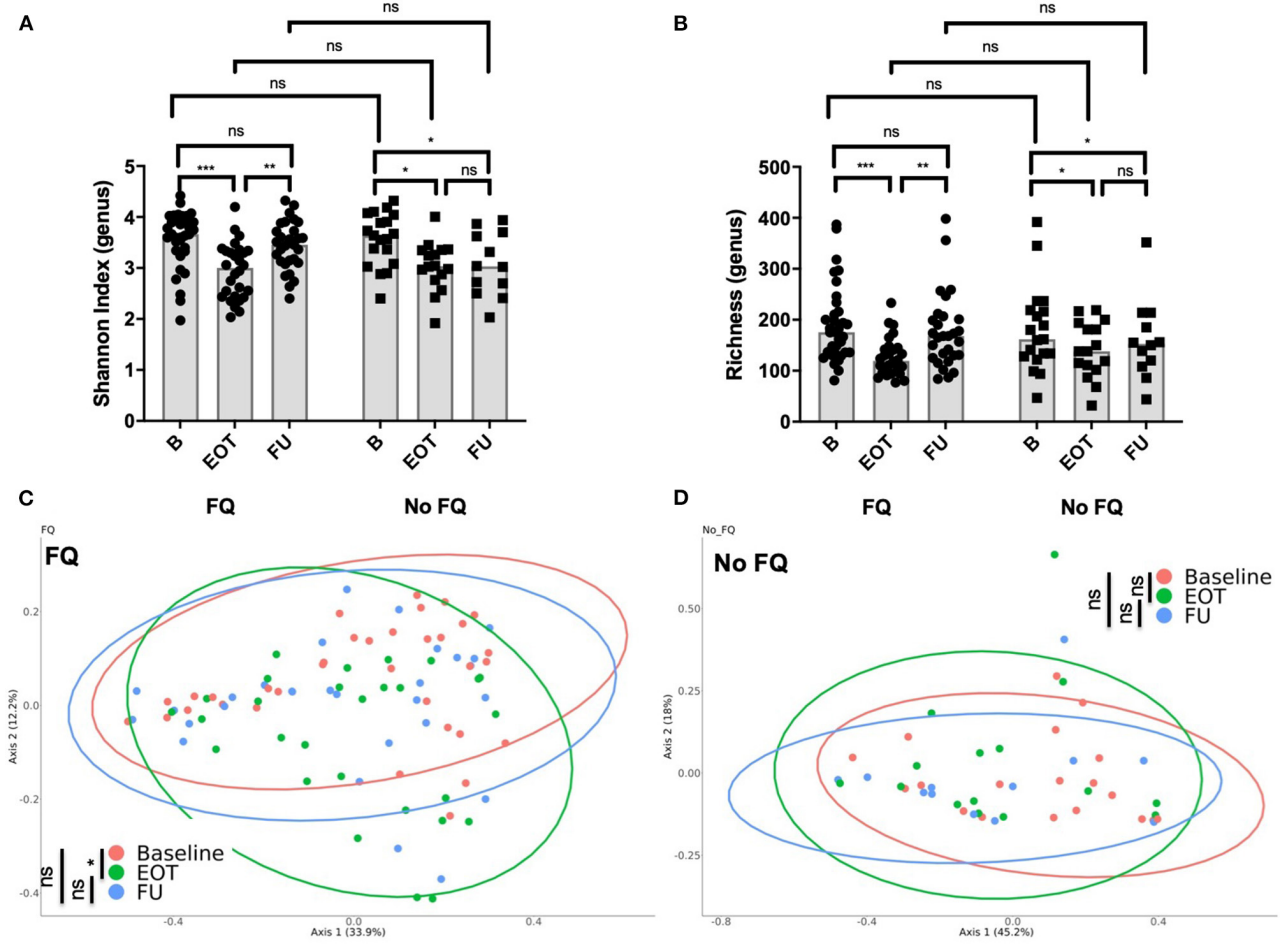
Impact of the use of fluoroquinolone (FQ) on gut microbiota composition. **(A)** Shannon index distribution (genus level) at the end of treatment and at follow-up (15 days after antibiotic withdrawal) according to exposition to FQ; Wilcoxon test for paired comparison and, Mann-Whitney test for inter-group comparisons. **(B)** Richness (genus level) at the end of treatment and at follow-up (15 days after antibiotic withdrawal) according to exposition to FQ; Wilcoxon test for paired comparison and, Mann-Whitney test for inter-group comparisons. Principal coordinate analysis of Bray–Curtis distance at three different times of sampling for **(C)** patients treated with FQ or **(D)** without FQ; permutational multivariate analysis of variance. B, baseline; EOT, end of treatment; FU, follow-up. *p < 0.05; **p < 0.01; ***p < 0.001.

The use of FQ was associated with a lower richness and Shannon index at the end of antibiotic treatment (Figures 4A,B). However, diversity rapidly recovered after FQ withdrawal, suggesting a high but transient impact of FQ on gut microbiota. Accordingly, the PCoA of the Bray–Curtis distance showed significant changes of the gut microbiota composition at the end of treatment in the FQ-treated group that recovered at FU, whereas no difference was found between baseline, EOT, and FU for patients that did not receive FQ (Figures 4C,D).

### Fecal Markers of Mucosal Inflammation Were Increased at the End of Treatment and Correlated With Microbiota Alpha-Diversity

**Figure 5:**
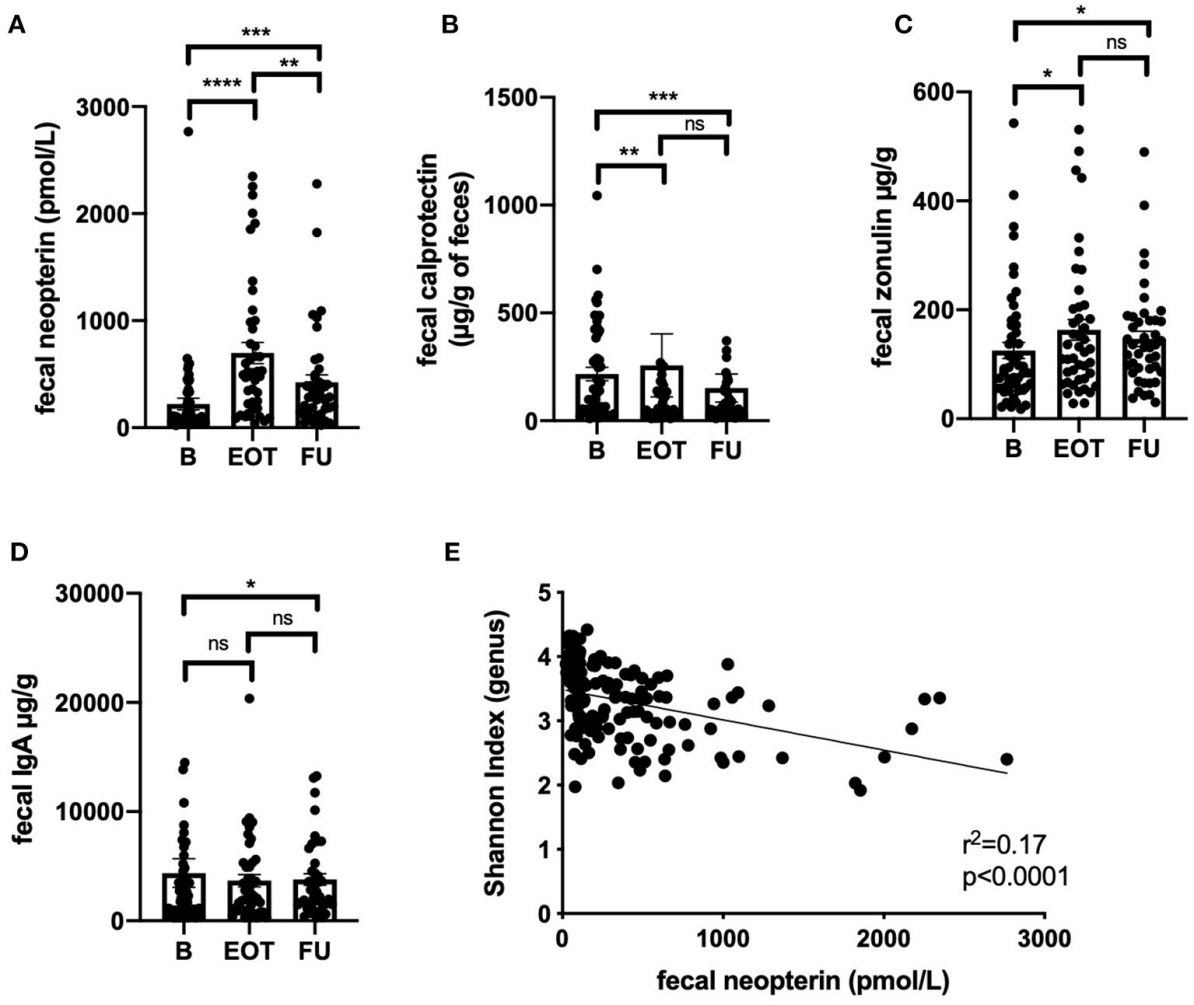
Correlation between markers of gut inflammation, permeability, and microbiota alpha-diversity. Values of fecal neopterin **(A)**, fecal calprotectin **(B)**, fecal zonulin **(C)**, fecal immunoglobulin A **(D)** at different time points; Wilcoxon test. **(E)** Correlation between fecal neopterin and the Shannon index (genus level) all along the study; simple linear regression. B, baseline; EOT, end of treatment; FU, follow-up. *p < 0.05; **p < 0.01; ***p < 0.001; ****p < 0.0001.

Fecal neopterin and fecal calprotectin, two markers of mucosal inflammation, were significantly increased at the end of treatment, with sustained changes that persisted at 2 weeks after antibiotic withdrawal (Figures 5A,B). However, only fecal neopterin reached a clinically relevant range of variation (≥200 pmol/g). Fecal zonulin also showed a significant increase at the end of treatment that persisted after 2 weeks, suggesting an increased intestinal permeability that lasted after exposure to antibiotics (Figure 5C). In line with a possible modification of mucosal immunity, fecal immunoglobulin A was also significantly modified at FU (Figure 5D). Strikingly, the level of fecal neopterin negatively correlated with the Shannon index all along the follow-up (Figure 5E, *r*^2^ = 0.17; *p* < 0.0001). The evolution of fecal markers in each subpopulation of BJI is detailed in Supplementary Figure 5.

### Systemic CRP Decreased With Antimicrobial Treatment, but Elevated CRP at EOT Correlated With Fecal Neopterin and Was Associated With a Distinct Gut Microbiota Composition

**Figure 6:**
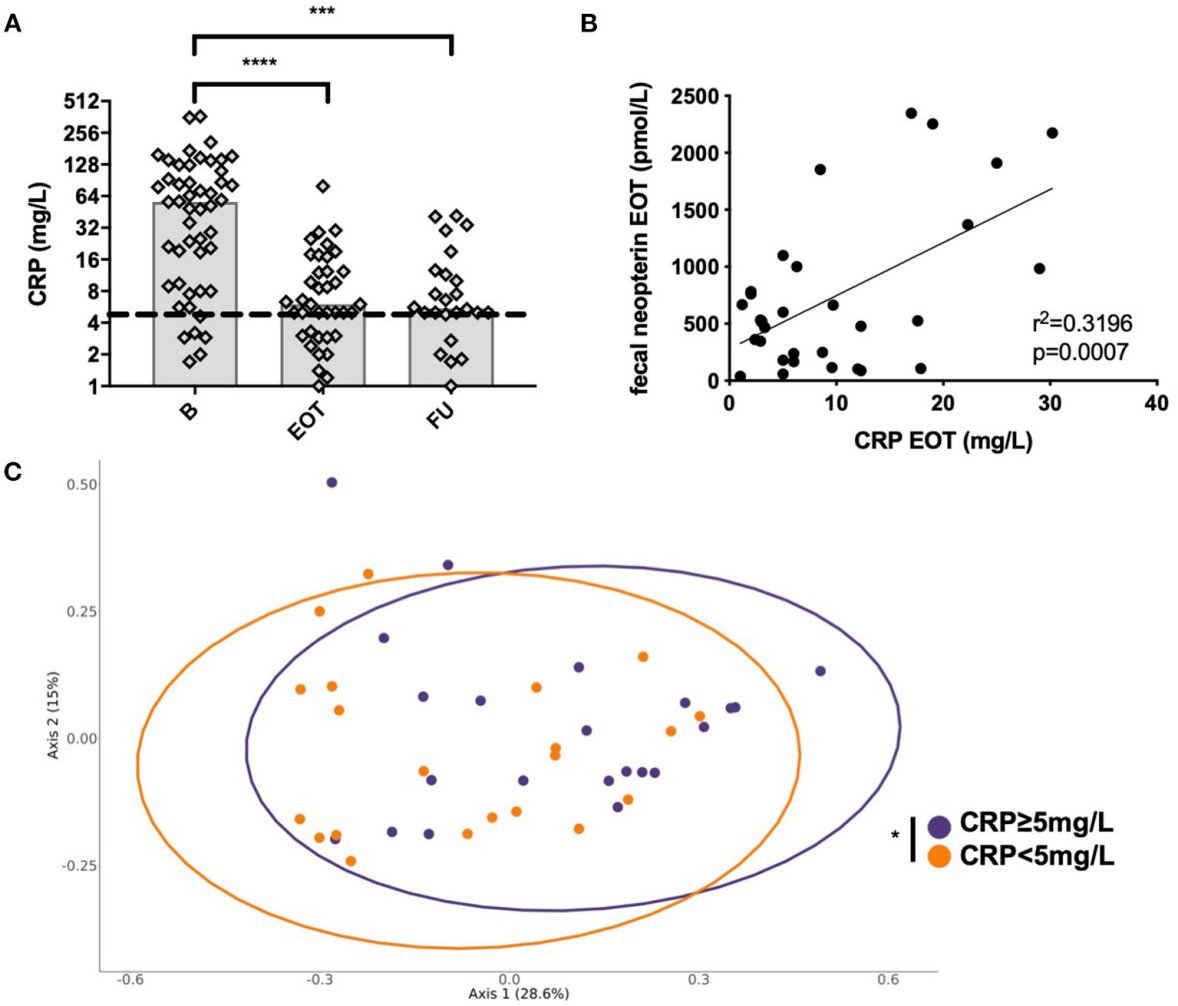
Correlation between systemic and mucosal markers of inflammation. **(A)** Evolution of the plasmatic level of C-reactive protein (CRP) at different time points. **(B)** Correlation between fecal neopterin and plasmatic CRP; simple linear regression. **(C)** Principal coordinate analysis of Bray–Curtis distance between patients with each sample colored according to plasmatic CRP level at the end of treatment; permutational multivariate analysis of variance. B, baseline; EOT, end of treatment; FU, follow-up. *p < 0.05; **p < 0.01; ***p < 0.001; ****p < 0.0001.

Systemic CRP significantly decreased from B to ETO with antimicrobial treatment and from B to FU (Figure 6A). As we hypothesized that residual systemic inflammation evaluated by CRP could be associated with microbiota alterations, we assessed the correlation between CRP and fecal neopterin, the markers of inflammation that varied the most at EOT. Of note is the fact that 28 patients had CRP ≥5 mg/L at EOT despite a favorable outcome of the BJI. We found that CRP at EOT correlated with fecal neopterin, and this suggests that residual systemic inflammation could be associated with gut inflammation rather than with a relapse of the BJI (Figure 6B). Moreover, the PCoA analysis of the Bray–Curtis distance showed that patients with elevated CRP at EOT presented a distinct gut microbial composition (PERMANOVA, *p* = 0.034) with an increase in *Fusobacterium* species (Figure 6C, Supplementary Figure 6).

### MDRB Fecal Carriage in the Fecal Microbiota of Patients Can Appear After Antibiotic Withdrawal

**Figure 7:**
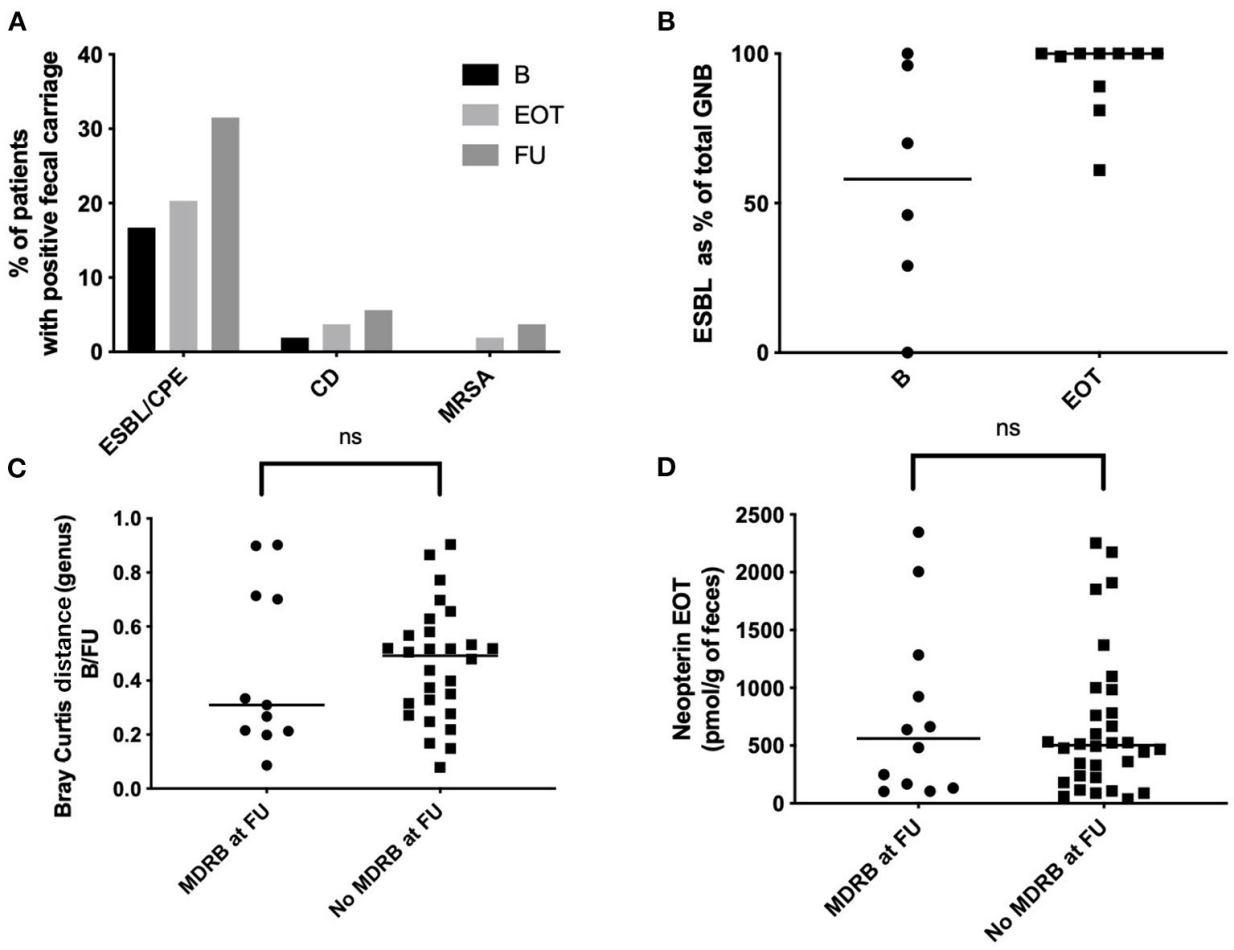
Fecal multi-drug-resistant bacteria (MDRB) and *Clostridioides difficile* carriage. **(A)** Proportion of patients with a positive fecal carriage (culture) at different time points. **(B)** EBSL as percentage of the total of all Gram-negative bacteria (aerobic culture). **(C)** Bray Curtis distance between baseline and 15 days after antibiotic withdrawal (FU) according to the carriage of MDRB; Mann–Whitney test. **(D)** Fecal neopterin at baseline and 15 days after antibiotic withdrawal (FU) according to the carriage of MDRB; Mann–Whitney test. B, baseline; EOT, end of treatment; FU, follow-up; ESBL, extended-spectrum beta-lactamases; MRSA, methicillin-resistant *Staphylococcus aureus*. *p < 0.05; **p < 0.01; ***p < 0.001.

Among the BJI population, nine patients were positive for MDRB fecal carriage at baseline (9 ESBL, 0 MRSA, 0 VRE; Figure 7A, Table 1). Among the 35 patients negative for MDRB at baseline and who performed MDRD screening at EOT, acquisition of ESBL was detected for six patients (6/35; 17.1%). Additional acquisition of MDRB at FU was observed for five patients (5/35, 14%: three ESBL, one carbapenem-resistant *Enterobacter*, and one rectal MRSA). Concerning *C. difficile*, one patient had an asymptomatic carriage at baseline and two other patients acquired C. *difficile* during the study (2/35, 5.7%). Overall, MDRB and *C. difficile* acquisition at EOT and FU represented 20% (7/35) and 37.1% (13/35) of all MDRB/*C*. *difficile*-free patients at the beginning of the study, respectively (Figure 7A). Of interest is that the quantification of MDRB-positive samples at FU in comparison to baseline clearly indicated an increased proportion of ESBL bacteria among fecal gram-negative bacteria (Figure 7B). Interestingly, among the nine patients with an ESBL carriage at baseline, five were still positive at EOT (5/9, 55.6%) and six at FU (6/9, 66.7%). MDRB carriage was not associated with differences in term of resilience (Bray–Curtis index between B and FU; Figure 7C) nor with different levels of fecal neopterin (Figure 7D).

## Discussion

This study explores, for the first time, how prolonged antibacterial therapy can disrupt the gut microbiota composition in the context of BJI. First, our data show that antibiotic treatment induced a significant loss of microbiota diversity that rapidly recovered at 2 weeks after the end of treatment for native and PJI but not for osteosynthesis-related BJI. These modifications were associated with distinct variations of bacterial phyla, in particular, with an increase of Proteobacteria and Bacteroidetes that did not fully recover at 2 weeks after antibiotic withdrawal. Second, comparing 6 to 12 weeks of antibiotic treatment did not show a major impact of treatment duration on the gut microbiota composition at the genus level and on microbiota diversity or resilience after treatment. In contrast, FQ was associated with a greater impact on microbiota diversity compared to other antibiotics, with a high resilience at FU. Third, fecal markers of inflammation were increased after antibiotic treatment, with a correlation between fecal neopterin and both microbial alpha-diversity and serum level of CRP. Moreover, patients with an elevated CRP presented a distinct gut microbial composition when compared to the others. This suggests that modifications of the gut microbiota by antibiotics could be associated with low-grade mucosal inflammation and residual systemic inflammation that possibly persisted at least 2 weeks after antibiotic withdrawal. Finally, as expected, antibiotic treatment was associated with MDRB acquisition and, particularly, ESBL emergence.

The potential effects of antibiotics on gut microbiota communities have been described for various treatments but only on small cohorts and rarely after prolonged antibiotic treatments (39). As observed in our work, loss of bacterial diversity was commonly reported particularly for molecules that target anaerobes with possible long-lasting effects even after a short course of antibiotic exposure (40). Indeed Jernberg and colleagues reported significant durable changes in *Bacteroides* clonal diversity up to 2 years after 7 days of clindamycin treatment (25), whereas others reported limited changes at 4 weeks after a short-term ciprofloxacin treatment (41).

Moreover, the effect of antibiotics on bacterial communities varies between individuals. For example, repetitive ciprofloxacin exposure amplifies microbial changes but only for some subjects (26). Thus, pre-treatment microbial diversity may account for differences in microbial communities' resilience and long-term effects of antibiotics. This initial dysbiosis/eubiosis state at the beginning of treatment may account for the rapid recovery of the gut microbial diversity of patients with native BJI compared to others. Indeed osteosynthesis-related BJI and PJI involved patients with complex infections and often previous antibiotic exposures. Even if the overall gut microbiota diversity can recover after treatment, definitive loss of some bacterial strains persists over time (21). Indeed, in our data, alpha-diversity seemed to be almost back to initial levels at 2 weeks after the end of treatment, but permanent changes in the abundance of specific species remained, of which the pathophysiological consequences remain unknown. However, functional redundancy supported by different bacterial species may counteract the possible effects of these permanent changes in microbiota composition (42).

When comparing the 6- and 12-week groups by PCoA analysis of the Bray–Curtis distance or when considering the correlation between antibiotic duration and alpha-diversity, the duration of treatment did not seem to affect the overall microbiota diversity or the resilience of the gut microbiota after antibiotic withdrawal. One possible explanation could be that 6 weeks of treatment is a sufficient amount of time to reach a microbial steady state that may persist with only small variations if antibiotics are prolonged. Indeed doses of antibiotics in BJI are, most of the time, high because of the limited diffusion into the bones of the majority of antibacterial therapies. This combination of high dose and prolonged exposure may favor a rather rapid achievement of a steady state of the gut microbiome that could be stable over time as the treatment is continued.

As described in numerous works, antibiotic exposure was associated with the selection of MDRB in our population. Notably in our cohort, the proportion of patients positive for MDRB or *C. difficile* reached almost 40% at FU. Even if MDRB decolonization is known to occur spontaneously after antibiotic withdrawal (43), increasing the amount of data suggests that genes for antibiotic resistance can persist for a long time once selected (44), which suggests a long-lasting effect of antibiotic treatement for patients with BJI.

One other striking result of this study is the identification of a correlation between markers of gut inflammation such as fecal neopterin and microbiota diversity. In rats, antibiotic exposure has already been associated, as we observed, with an increase of gut permeability and increased plasma levels of haptoglobin, a precursor of zonulin (45). Interestingly, the authors reported similar modifications of the gut microbiota with an increase of Proteobacteria and a general decrease of microbiota diversity. In the same line, Feng et al. (27) reported similar modifications of gut permeability after antibiotic treatment in mice, associated with the activation of the NLRP3 inflammasome and autophagy.

We also found that residual systemic inflammation evaluated by CRP correlated with fecal neopterin and, in consequence, with the potential persistence of microbiota alterations at the end of antibiotic treatment. It is of importance, as CRP is usually monitored to evaluate the BJI's response to antibiotics. Indeed some physicians consider that if CRP is still elevated at the end of treatment, it could be due to bacterial persistence at the site of bone infection, leading to consideration of prolongation of the antibiotic treatment. However, data indicate that the CRP level at the end of treatment is not predictive of a persistent infection (46, 47). Thus, our results raise the hypothesis that abnormal CRP at the end of the treatment could be a potential marker of gut barrier dysfunction associated with microbial dysbiosis. Further data are required to confirm this hypothesis.

Antibiotic impact on the gut microbiota has potential long-term effects which suggest several measures to correct or prevent these changes. The best way would be to minimize the use of antibiotics, preferentially by using *in situ* antibiotics or using antibiotics with a narrow spectrum to limit the impact on the gut microbiota. However, in many infections such as BJI, antibiotics cannot be replaced, and long-term systemic treatment at high dosage is mandatory to cure patients. In these situations, microbe-based therapy to counteract the deleterious ecological effects of such treatments could be of interest, especially in selected populations at risk of non-recovery (elderly, personal medical history of *C. difficile* infection, low microbiota diversity, possibly osteosynthesis-related BJI, etc.). When specifically targeting the gut microbiota, different tools are commonly used. Either selected microbes (bacteria and/or fungi) can be added as probiotics or specific molecules (prebiotics) can be used to promote specific species of interest, but as the gut microbiota is a complex ecosystem, such tools may miss significant network interactions at the level of bacterial species or between different kingdoms (bacteriophages and fungi, for example).

Moreover, there are some concerns about probiotics, as they are composed of only a few bacterial species, and their capacity to positively impact antibiotic-associated dysbiosis is debatable.

Fecal microbiota transplantation is nowadays the only treatment that permits the engraftment of a complex ecosystem with proven functional benefits. It consists of the transfer of the fecal microbial ecosystem of a healthy donor to a recipient in order to restore gut homeostasis. Evaluation of fecal microbiota transplantation in various pathological conditions is now blooming, with contrasting results extending the need to validate the administration modality and long-term safety (48).

Our study has some limitations. First, the relatively low number of evaluated patients may account for a lack of power especially in subgroup analysis. Moreover, some patients did not perform stool sampling at all time points, which may also have induced some bias. However, it is, to our knowledge, one of the largest clinical studies evaluating the effects of prolonged antimicrobial therapy on the gut microbiota. The use of rectal swabs may facilitate recruitment and increase patient adherence to develop larger studies. Furthermore, evaluation of microbial composition and inflammatory markers at a more distant time point after antibiotic withdrawal would be of great interest to assess the long-term impact of antibiotics on the gut ecosystem and mucosal physiology.

In conclusion, to our knowledge, this is the first study that explores the impact of prolonged antibiotic treatment on gut microbiota in the context of BJI. As expected, antibiotics significantly altered the gut microbiota diversity and composition, with a rapid but partial recovery observed at 2 weeks after antibiotic withdrawal. Antibiotic duration or the use of FQ did not seem to affect this resilience. These modifications were associated with an increase in markers of mucosal inflammation and gut permeability and elevated levels of CRP. Further studies are needed to explore these possible links and their impact on resilience. Finally, as illustrated in our cohort, acquisition of MDRB remains one the most challenging side effects of long-term exposure to antibiotics. Innovative microbe-based therapies could be a promising tool to address these issues.

## Data Availability Statement

The datasets presented in this article are not readily available because of legal reasons. Requests to access the datasets should be directed to the corresponding author.

## Ethics Statement

The studies involving human participants were reviewed and approved by Comité de Protection des Personnes SUD-EST II. Written informed consent to participate in this study was provided by the participants' legal guardian/next of kin.

## Author Contributions

TF designed the study with BL and CC. TF, CB, SL, CP, DB, F-AD, VZ, ES, TF, and CC managed the patients. JJ and FL generated resistances data. MM and T-TL managed the centralization and biobanking of samples. BL, NB, and TF performed the literature review and wrote the first draft of the manuscript. BL, NB, and CG performed the data analysis. All authors contributed to the improvement of the manuscript.

## Conflict of Interest

BL and CG are employed by the commercial company MaaT Pharma. LB was employed by the commercial company MaaT Pharma. NB declared a travel grant from Maat. JD is co-founder of MaaT Pharma. TF received advisory honorarium from MaaT Pharma and was the principal investigator of this study. The remaining authors declare that the research was conducted in the absence of any commercial or financial relationships that could be construed as a potential conflict of interest. The authors declare that this study received funding from MaaT Pharma. The funder had the following involvement in the study: design, management and analysis.

## Supplementary Material

Supplementary Figure 1 Richness evaluated at each time of sampling according to the type of bone joint infection. Wilcoxon test for paired comparison and Mann–Whitney analysis for inter-group comparisons were used. B, baseline; EOT, end of treatment; FU, follow-up.

Supplementary Figure 2 Bacterial species that vary the most after antibiotic treatment. Variations correspond to the ratio of relative abundance between **(A)** baseline and the end of treatment and **(B)** between baseline and follow-up (15 days after antibiotic withdrawal). Descriptions of the 20 bacteria that varied the most in relative abundance between B and EOT and B and FU for each group of patients are presented. B, baseline; EOT, end of treatment; FU, follow-up.

Supplementary Figure 3 Linear discriminant analysis effect size showing species that support differences between baseline and end of treatment.

Supplementary Figure 4 Correlation between the Shannon index and clinical parameters. Correlation between the age at baseline and the Shannon index at baseline **(A)**, end of treatment **(B)**, and 15 days after antibiotic withdrawal **(C)**. Correlation between the body mass index at baseline and the Shannon index at baseline **(D)**, end of treatment **(E)**, and 15 days after antibiotic withdrawal **(F)**. *r*, Pearson correlation coefficient; B, baseline; EOT, end of treatment; FU, follow-up.

Supplementary Figure 5 Correlation between markers of gut inflammation, permeability, and microbiota alpha-diversity according to the type of bone joint infection. Values of fecal neopterin **(A)**, fecal calprotectin **(B)**, fecal zonulin **(C)**, and fecal immunoglobulin A **(D)** at different time points; Wilcoxon test. B, baseline; EOT, end of treatment; FU, follow-up.

Supplementary Figure 6 Linear discriminant analysis effect size showing species that support differences between patients with an elevated C-reactive protein (≥5 mg/L) at the end of treatment and others.
