# Supplementary figures and images for: Impact on the Gut Microbiota of Intensive and Prolonged Antimicrobial Therapy in Patients With Bone and Joint Infection
Source: Front Med (Lausanne). 2021 Mar 5;8:586875. doi: 10.3389/fmed.2021.586875 (PMC7977441; doi:10.3389/fmed.2021.586875)

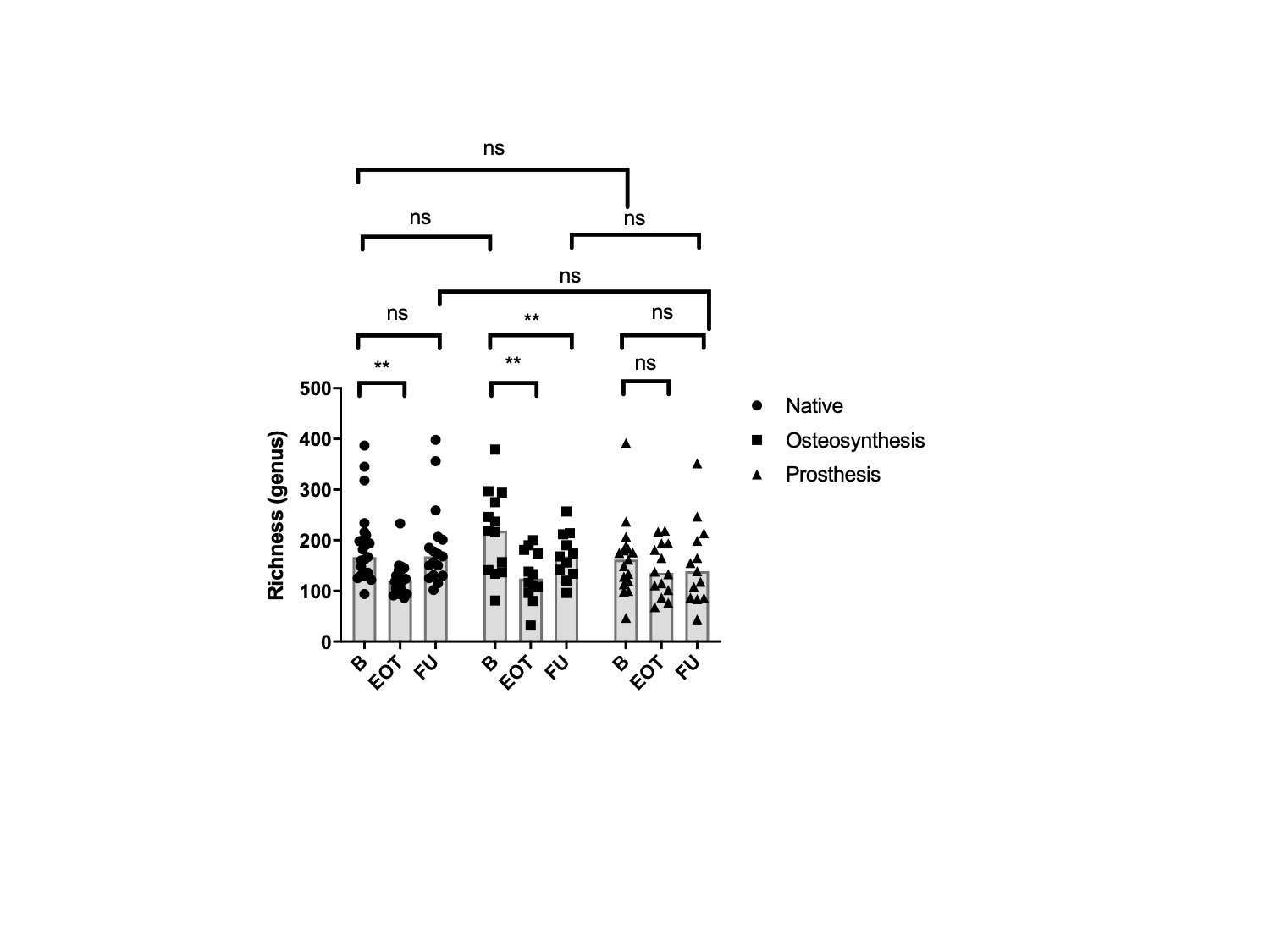

Supplement: Supplementary Figure 1 — Richness evaluated at each time of sampling according to the type of bone joint infection. Wilcoxon test for paired comparison and Mann–Whitney analysis for inter-group comparisons were used. B, baseline; EOT, end of treatment; FU, follow-up. [file Image_1.JPEG]

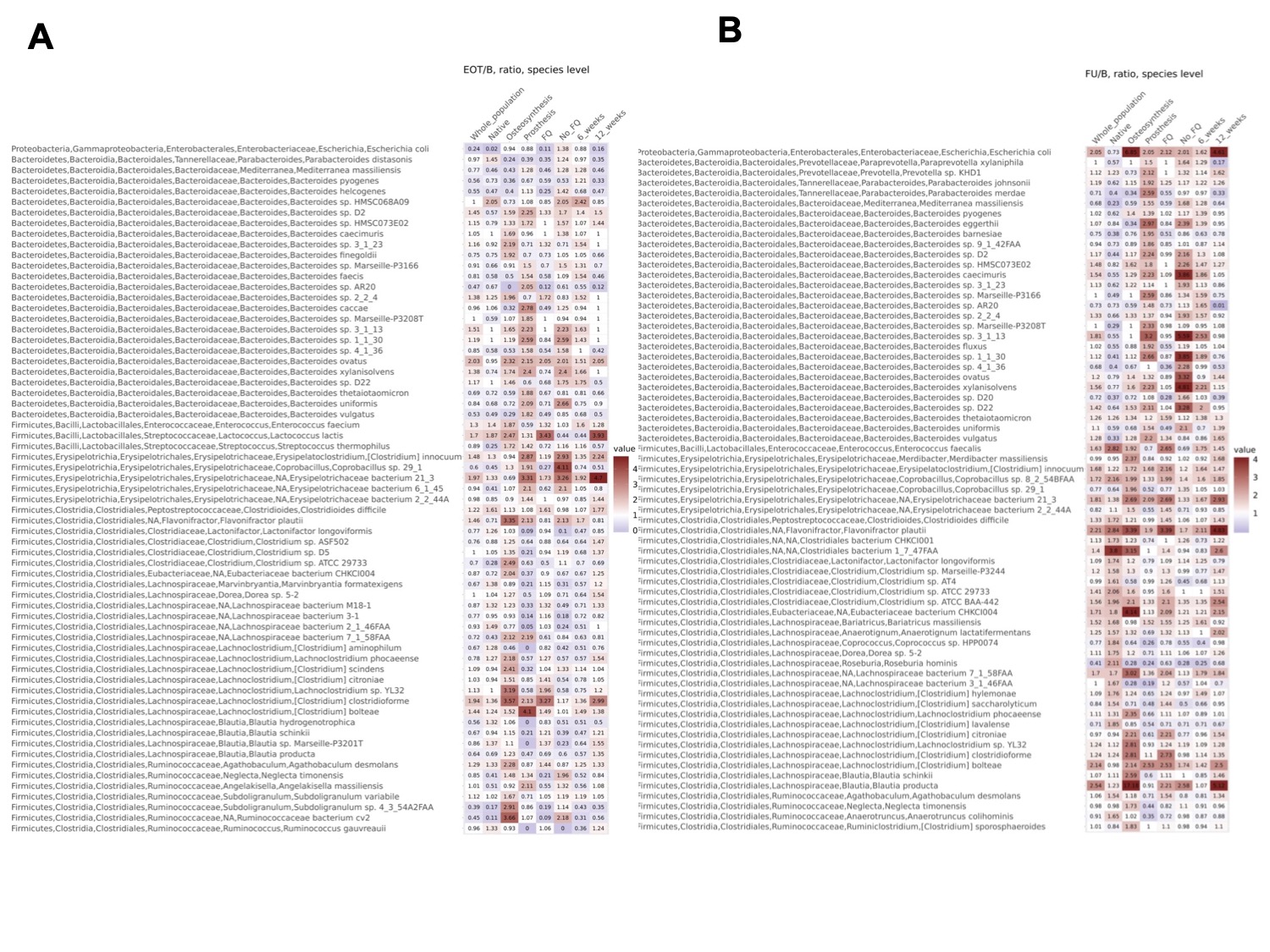

Supplement: Supplementary Figure 2 — Bacterial species that vary the most after antibiotic treatment. Variations correspond to the ratio of relative abundance between (A) baseline and the end of treatment and (B) between baseline and follow-up (15 days after antibiotic withdrawal). Descriptions of the 20 bacteria that varied the most in relative abundance between B and EOT and B and FU for each group of patients are presented. B, baseline; EOT, end of treatment; FU, follow-up. [file Image_2.JPEG]

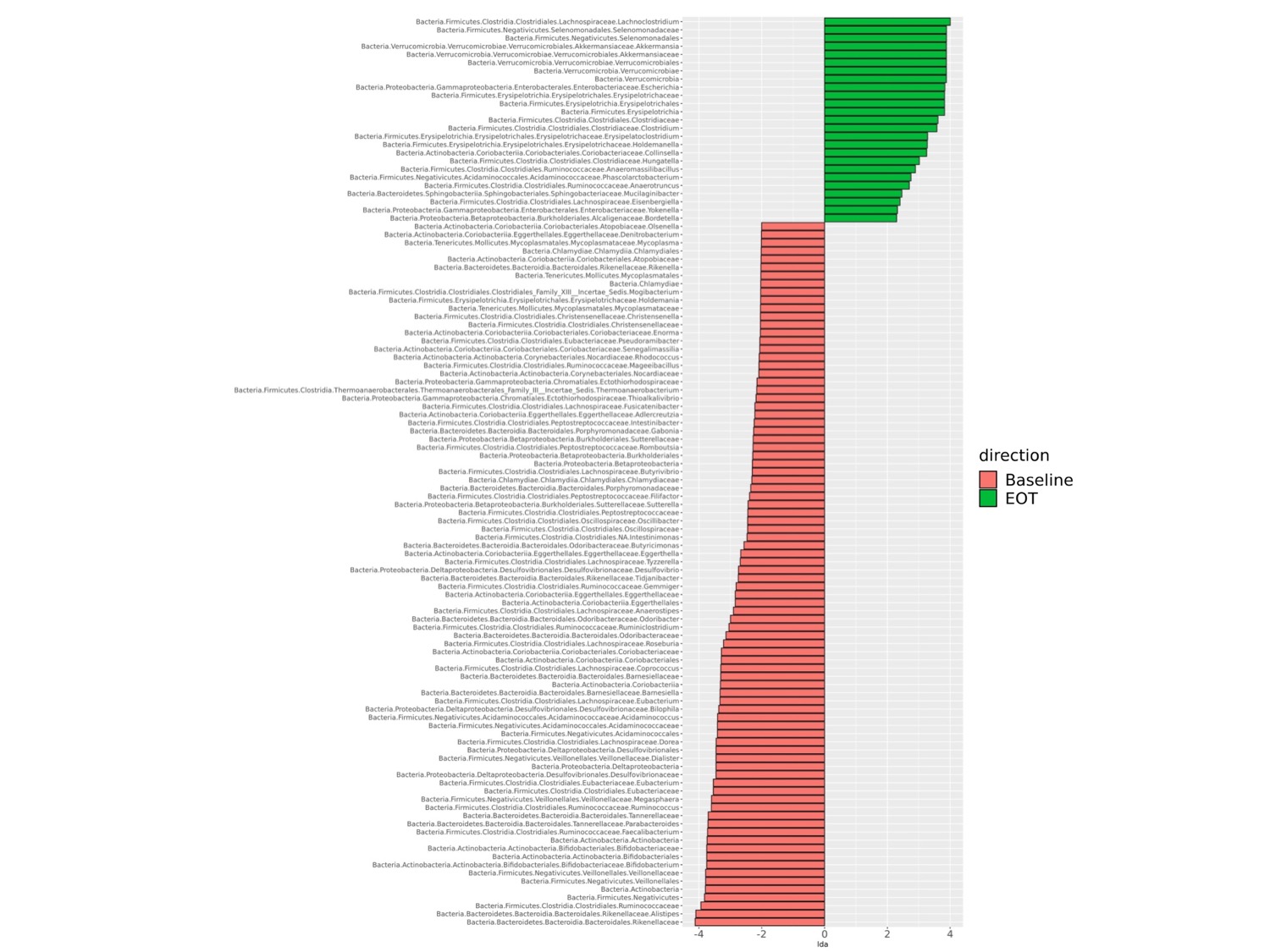

Supplement: Supplementary Figure 3 — Linear discriminant analysis effect size showing species that support differences between baseline and end of treatment. [file Image_3.JPEG]

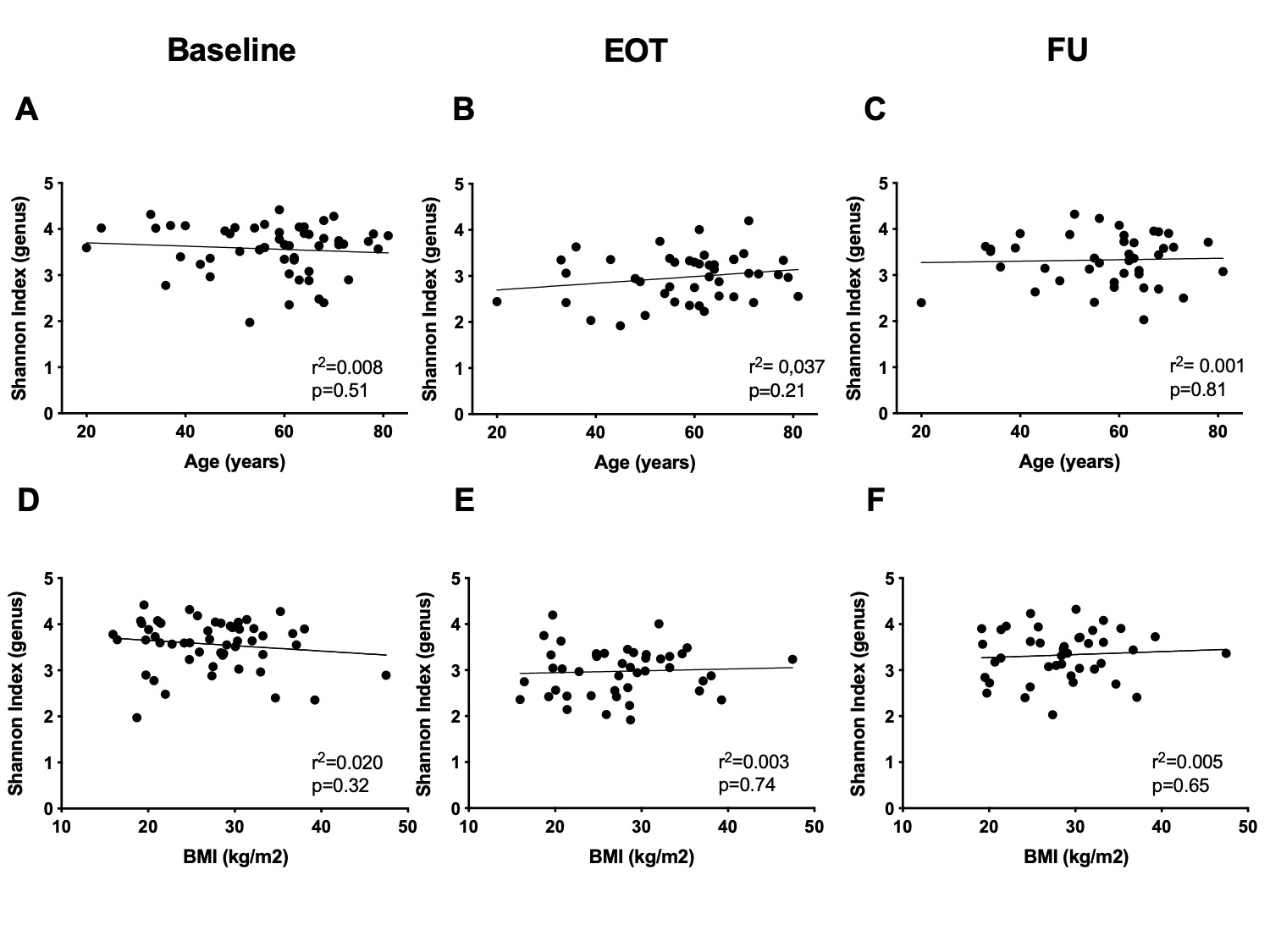

Supplement: Supplementary Figure 4 — Correlation between the Shannon index and clinical parameters. Correlation between the age at baseline and the Shannon index at baseline (A), end of treatment (B), and 15 days after antibiotic withdrawal (C). Correlation between the body mass index at baseline and the Shannon index at baseline (D), end of treatment (E), and 15 days after antibiotic withdrawal (F). r, Pearson correlation coefficient; B, baseline; EOT, end of treatment; FU, follow-up. [file Image_4.JPEG]

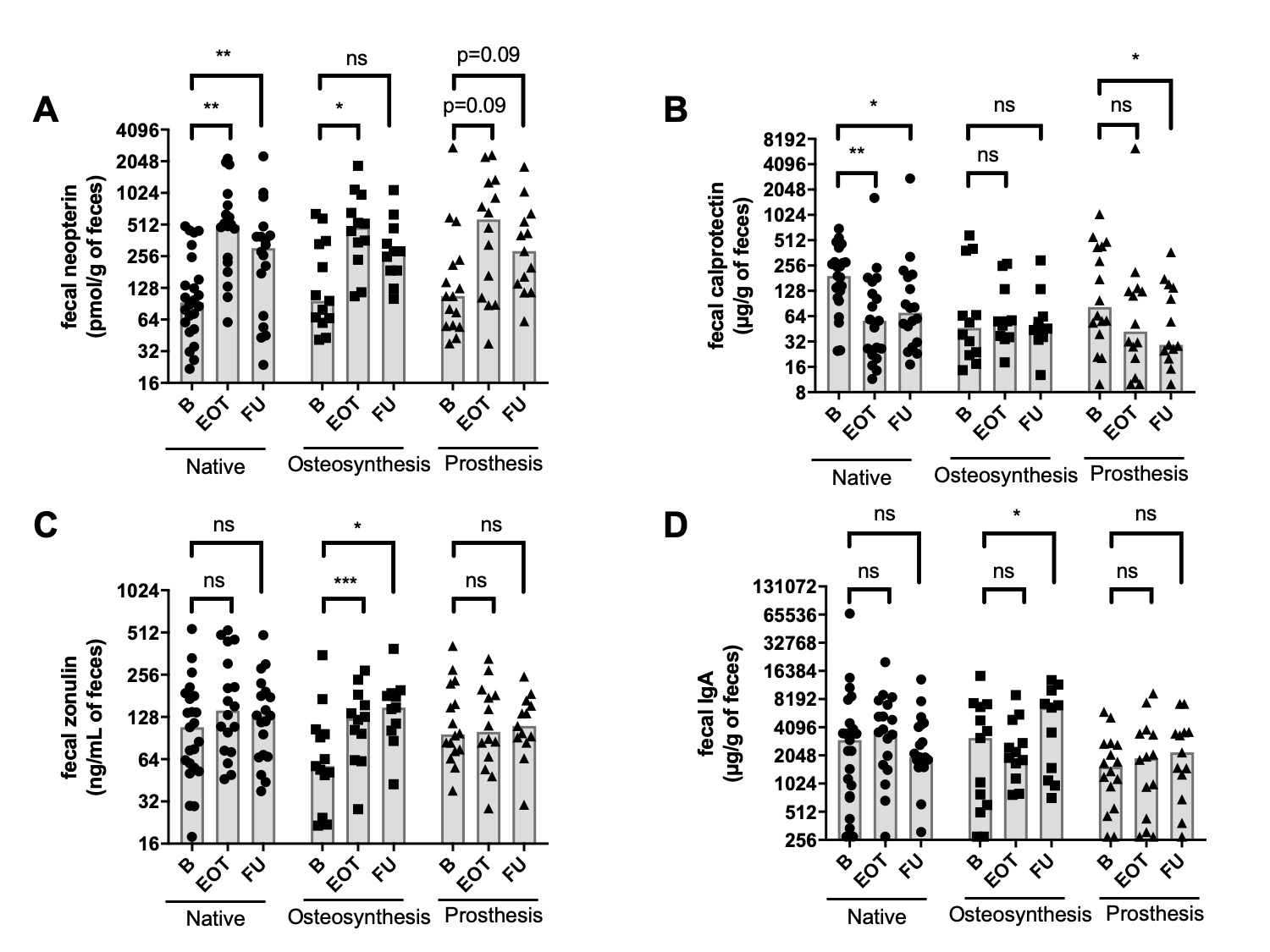

Supplement: Supplementary Figure 5 — Correlation between markers of gut inflammation, permeability, and microbiota alpha-diversity according to the type of bone joint infection. Values of fecal neopterin (A), fecal calprotectin (B), fecal zonulin (C), and fecal immunoglobulin A (D) at different time points; Wilcoxon test. B, baseline; EOT, end of treatment; FU, follow-up. [file Image_5.JPEG]

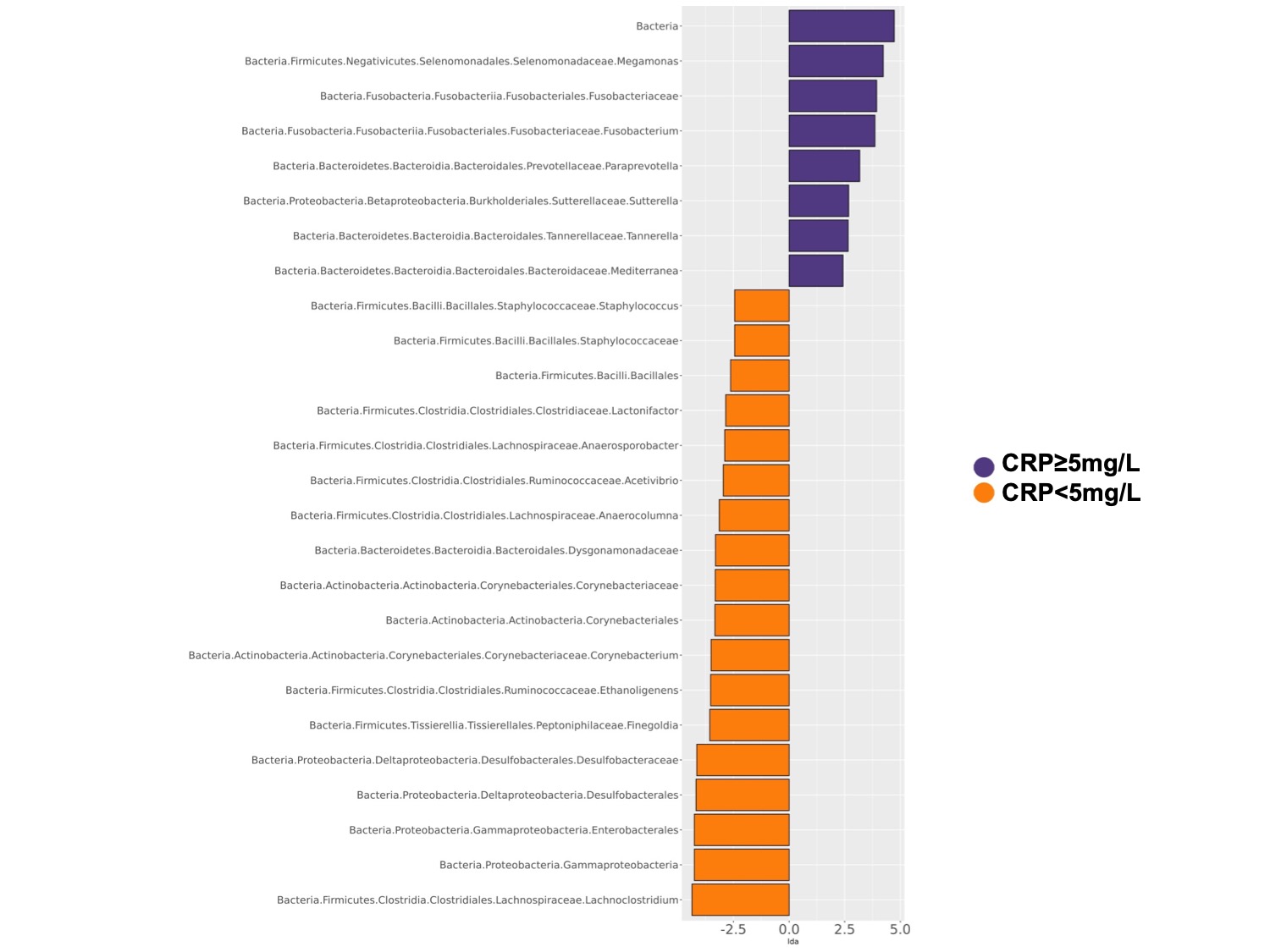

Supplement: Supplementary Figure 6 — Linear discriminant analysis effect size showing species that support differences between patients with an elevated C-reactive protein (≥5 mg/L) at the end of treatment and others. [file Image_6.JPEG]
